# Gestational Diabetes Mellitus and Energy-Dense Diet: What Is the Role of the Insulin/IGF Axis?

**DOI:** 10.3389/fendo.2022.916042

**Published:** 2022-06-23

**Authors:** Irene Martín-Estal, Fabiola Castorena-Torres

**Affiliations:** Tecnologico de Monterrey, Escuela de Medicina y Ciencias de la Salud, Monterrey, Mexico

**Keywords:** IGF-1 (insulin-like growth factor-1), energy-dense diet, obesity, gestational diabetes mellitus (GDM), placenta

## Abstract

Gestational diabetes mellitus (GDM), is one of the most important pregnancy complications affecting approximately 15% of pregnant women. It is related to several gestational adverse outcomes in the fetus, *e.g.*, macrosomia, shoulder dystocia, stillbirth, neonatal hypoglycemia, and respiratory distress. Women with GDM have a high risk of developing type 2 diabetes in the future. The pathogenesis of GDM is not completely understood; nevertheless, two factors could contribute to its development: β-cell dysfunction and failure in insulin secretion in response to insulin resistance induced by gestation. Both processes, together with the physiological activities of the insulin-like growth factors (IGFs), play a crucial role in glucose transport to the fetus and hence, fetal growth and development. IGFs (both IGF-1 and IGF-2) and their binding proteins (IGFBPs) regulate glucose metabolism and insulin sensitivity. Maternal nutritional status determines the health of the newborn, as it has substantial effects on fetal growth and development. Maternal obesity and an energy-dense diet can cause an increase in insulin and IGF-1 serum levels, producing metabolic disorders, such as insulin resistance, GDM, and high birth weight (> 4,000 g) due to a higher level of body fat. In this way, in GDM pregnancies there is an increase in IGF-1 and IGF-2 serum levels, and a decrease in IGFBP-1 and 4 serum levels, suggesting the crucial role of the insulin/IGF system in this gestational outcome. Here, the present review tries to elucidate the role that energy-dense diets and the insulin/IGF-1 signaling pathway perform in GDM pregnancies.

## Introduction

According to the World Health Organization (WHO), malnutrition refers to deficiencies, excesses or imbalances in a person’s intake of energy and/or nutrients, leading to undernutrition or overnutrition (1). The increasing prevalence of obesity has implications for the health of human population as this condition augments the risk of developing several serious diseases (2). Overweight and obesity rates increased by two-fifths between 1990 and 2010, especially in women of reproductive age (3, 4), being a highly prevalent pathology in Latin American countries (> 30%) (5, 6). Therefore, obesity represents an enormous threat to public health (7, 8).

Maternal obesity before and after conception increases the risk of a wide range of pregnancy-related complications (9). Experimental animal studies have shown that obesity during gestation impairs glucose tolerance, promotes insulin resistance, endothelial cell dysfunction, hypertension, hyperphagia and increases adiposity in offspring (10, 11). Moreover, obesity during pregnancy can lead to gestational diabetes mellitus (GDM), an adverse condition that increases the risk of fetal overgrowth (macrosomia), fetal adiposity and several alterations throughout infant’s life, including predisposition to obesity, type 2 diabetes mellitus (T2DM) and metabolic disorders (7, 12). As it is shown in several clinical studies, GDM has been associated with high concentrations of numerous hormones, such as insulin-like growth factor 1 (IGF-1, an essential hormone for intrauterine and postnatal growth and development), insulin; and other molecules with endogenous functions, *e.g.*, glucose, C-reactive protein, fibrinogen, lipids, etc. (13). Particularly, a recent systematic review summarizes the clinical studies of GDM where some molecular biomarkers of IGF-1 signaling pathway have been analyzed; but the existing evidence is inconclusive, so it is necessary to elucidate this mechanism (14).

The main reason for this rise in obesity, and thus diabetes and GDM problems, is due to the consumption of foods and/or diets rich in fats and sugars, which may be attributable to alterations in the insulin/IGF-1 signaling pathway (15). However, the relationship between this cascade and pregnancy adverse outcomes is not entirely known.

## Energy-Dense (High Intake of Sugars and Saturated Fatty Acids) Diet in Pregnancy

Nowadays, energy-dense diets are a constant trend in society, due to the accessibility of their products, both in large and small commerces. In the Western world these diets are regularly consumed during pregnancy (16). These diets are characterized by an elevated intake of sugars and fatty acids, foods that have a high energy density (4 kcal/g and 9 kcal/g, respectively), defined as the amount of energy in a particular weight of food (17, 18).

Due to its main functions in growth, IGF-1, as well as growth hormone (GH), nutrients are an important part of its signaling transduction regulation. In this sense, clinical and experimental studies have shown that intake of protein (especially from milk and yogurt), fibre, starch from wholegrains, redmeats, fats and oils are positively associated with IGF-1 serum levels (19–21). The disparity of one or more nutrients could affect growth, anabolism and nutrient sensing.

In addition to the increased availability of high density foods, the decreased needs for physical exertion have promoted the raise in obesity before, during and after pregnancy. Clinical studies have shown that overweight women during pregnancy have higher insulin and IGF-1 levels, which can have substantial impact on women and fetal health (15). For example, maternal malnutrition during pregnancy can lead to fetal growth restriction (FGR), an IGF-1 deficiency condition characterized by a low neonatal birth weight (< 2,500 g) (22). Conversely, maternal obesity before gestation, excessive weight gain during pregnancy or an energy-dense diet (high intake of sugars and saturated fatty acids) can promote high birth weight (> 4,000 g), the development of insulin resistance and GDM during pregnancy, and the incidence of metabolic disorders and high deposition of body fat in children born from obese mothers (15, 23–25).

Experimental models of high fat diets have shown hyperphagia in the offspring from mothers fed with sugar high diets, but not high in fat or low in carbohydrates (10), preferring high-fat, sugary and salty foods rather than normal chow diet (26, 27). Moreover, these high-sugar diets and a combination of being overweight/obese before pregnancy and/or junk food diet during this period may increase the risk of macrosomia and overweight in newborns and in later life (28–30).

Likewise, energy-dense diets reduce glucose tolerance, alter insulin sensitivity in late pregnancy and feto-placental glucose metabolism, as insulin/IGF signaling is impaired, leading to maternal metabolic dysfunction that can have several consequences for fetal growth (16, 30). For example, experimental studies have disclosed that energy-dense diets promote fetal hepatic steatosis, due to an increase in circulating triglycerides, and hypoxemia, increasing amino acid metabolism for energy production in fetal liver (31, 32). Furthermore, clinical studies disclosed that low adherence to the Mediterranean diet (characterized by the consumption of a high intake of extra virgin oil, fruits, cereals, legumes, vegetables; and a moderate/low intake of fish, seafood, eggs, meat and dairy products) is associated with an altered GH/IGF-1 response, resulting in a poor body composition and cardiometabolic profile (21, 33).

Furthermore, regardless of maternal obesity, high-fat diets and excess of energy-dense diet intake throughout pregnancy can result in placental alterations in morphology and/or function. This increases inflammation and fatty acid transport, that could permanently alter offspring physiology (27), promoting adiposity, adult hyperinsulinemia, hyperleptinemia, and the development of T2DM and cardiovascular diseases (11, 34–36).

## Gestational Diabetes Mellitus (GDM)

GMD is the most prevalent metabolic disorder during pregnancy, diagnosed in the second or third trimesters with high blood glucose levels, frequently disappearing after delivery, where glucose tolerance is restored to normal levels. An excessive gestational weight gain in the first trimester of pregnancy might denote a serious period for GDM development (12).

Predominantly, the second trimester of gestation is a period where insulin sensitivity is impaired, in order to limit maternal glucose uptake to maintain a suitable nutrient supply for the growing fetus (37, 38). This could be due to the effects of placental hormones, *e.g.*, placental lactogen (PL) and GH, which stimulate the liver increasing growth factor levels, including IGF-1 (39). It could also be a result of a normal augment in maternal adiposity, as lipolysis and free fatty acids metabolism are promoted, causing compensatory hyperinsulinemia that increases adipogenesis, inflammatory adipokines and insulin resistance (40).

GDM increases the development of maternal, fetal and neonatal complications. It is related to numerous gestational difficulties, such as placental vasculature alterations, macrosomia, shoulder dystocia, stillbirth, neonatal hypoglycemia and respiratory distress (41). This disorder could be a risk factor for T2DM, metabolic and cardiovascular disorders development in the mother and her offspring in later life (13, 42–44).

Although the pathogenesis of GDM is still unknown, two contributing factors have been involved in its progression: β-cell dysfunction and alterations in insulin secretion to compensate for insulin resistance induced by pregnancy (45, 46). Also, it has been observed in animal experimental models and clinical studies that obesity during pregnancy, besides promoting insulin resistance, can impair glucose tolerance, increase IGF-1 serum levels, reduce insulin-like growth factor binding proteins (IGFBPs), and endorse endothelial cell dysfunction, hypertension, hyperphagia and increased adiposity in offspring (10, 11, 47–49).

GDM is associated with fetal hyperglycemia and hyperinsulinemia, which in turn lead to feto-placental endothelial dysfunction, at both macro and microvasculature levels, similar to that found in adult T2DM patients (50). Also, GDM pregnancies exhibit alterations in nitric oxide (NO) bioavailability (51) and other vasoactive molecules (*e.g.*, adenosine) and/or differential responses to hormones (*e.g.*, insulin, vascular endothelial growth factor -VEGF-) (51–54), that can result in distorted angiogenesis and hence, the aforementioned endothelial dysfunction (51, 55). This endothelial dysfunction, known as the diminished ability of the placenta to stimulate vasodilation, can involve signaling mechanisms from the disease itself or adaptative responses to the abnormal intrauterine environment (56).

Glucose is the primary metabolic fuel for the fetus (50-80%), the amniotic fluid is the second largest source for this metabolite *via* fetal swallowing (10-15%) (57–59). In this sense, metabolites and hormones in amniotic fluid play an important role in fetal development. For example, in GDM pregnancies there is a decrease in IGFBP-1 levels and an increase in glucose and insulin levels in amniotic fluid, leading to an intrauterine exposure to glucose that accelerate the exhaustion of β-cells, a characteristic effect of GDM (59, 60).

## Role of the Insulin/IGF Axis in GDM

The insulin-like growth factor (IGF) system is conformed of two growth factors, IGF-1 and IGF-2; three receptors, IGF-1 receptor (IGF1R), IGF-2 receptor (IGF2R) and hybrid receptor; and IGFBPs (61). This system is involved in growth, particularly during fetal development, metabolism and crucial cellular processes such as proliferation, survival, cell migration and differentiation (62).

As aforementioned, the production of IGF-1 is dependent on a suitable supply of nutrients, such as glucose, amino acids and lipids. It is secreted in practically every tissue for autocrine and/or paracrine purposes (63). GH is responsible for stimulating IGF-1 secretion, forming the GH/IGF-1 axis, where GH secretion is promoted by growth hormone-releasing hormone (GHRH) and inhibited by somatostatin. Herein, IGF-1 can inhibit GH expression by stimulating somatostatin secretion, thus hindering GH secretion (64).

**Figure 1:**
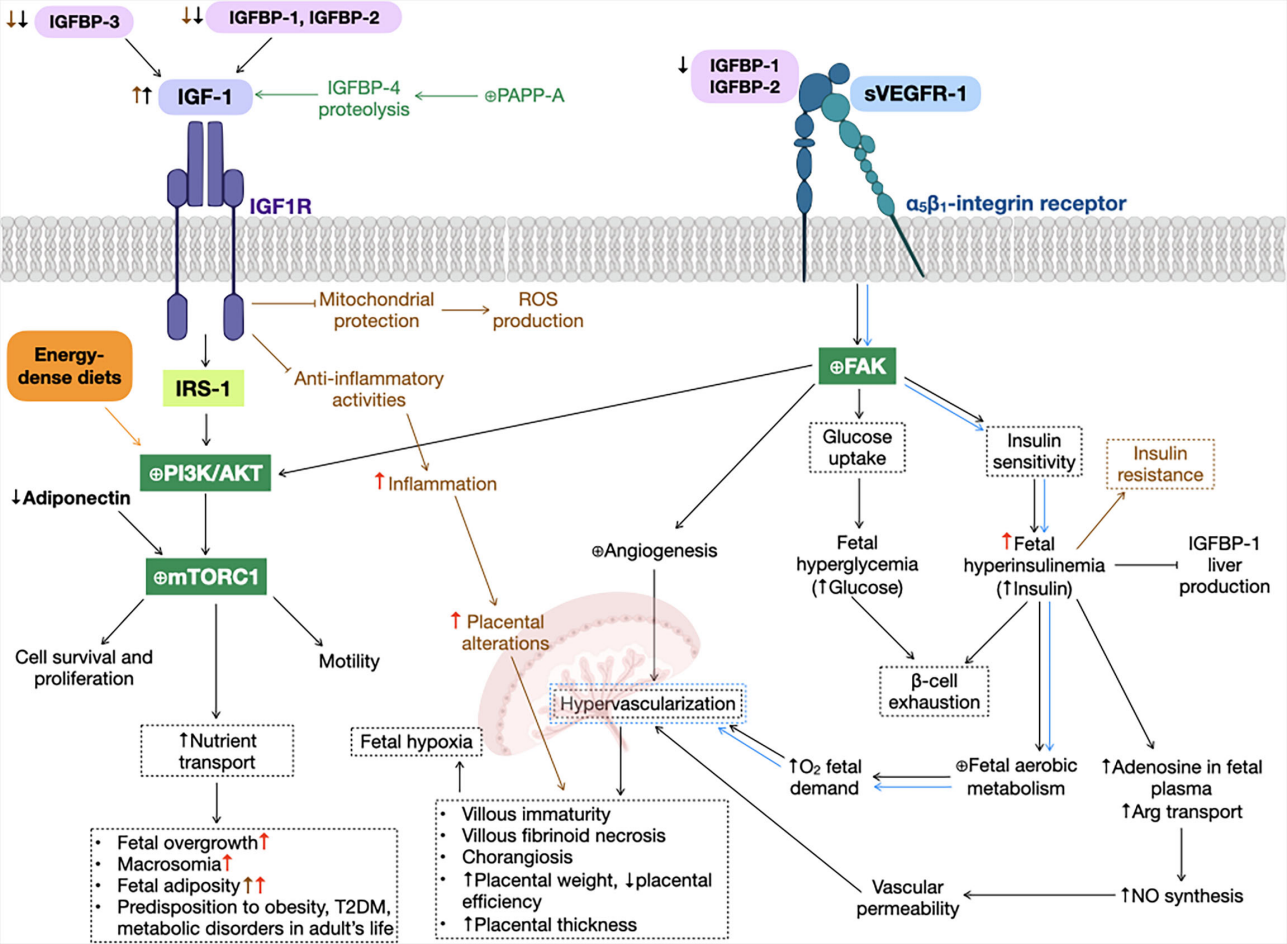
Gestational diabetes mellitus (GDM) alterations in insulin-like growth factor 1 (IGF-1) signaling pathway. GDM (black arrows) and preeclampsia (green text and arrows) reduce insulin-like growth factor binding protein 1 (IGFBP-1) levels, increasing IGF-1 bioavailability, that promotes phosphatidylinositol-3-kinase (PI3K)/protein kinase B (AKT) cascade (as well as energy-dense diets, orange text and arrows), activating mammalian target of rapamycin complex 1 (mTORC1), that results in cell survival and proliferation, motility and an augment nutrient transport, leading to fetal overgrowth, macrosomia, fetal adiposity, type 2 diabetes mellitus (T2DM), metabolic disorders and predisposition to obesity in adult life. Additionally, soluble form of the vascular endothelial growth factor receptor 1 (sVEGFR-1) can bind to α_5_β_1_-integrin receptor (blue arrows), expressed predominantly in extravillous trophoblasts, activating the focal adhesion kinase (FAK), promoting fetal hyperglycemia and hyperinsulinemia, that lead to β-cell exhaustion, a principal characteristic of GDM. This increase in insulin levels cause promote nitric oxide (NO) synthesis and fetal aerobic metabolism, both producing hypervascularization in the placenta, that could result in several physiological alterations in this organ, such as villous immaturity, villous fibroid necrosis, chorangiosis, increased placental weight and thickness, and decreased placental efficiency. Also, FAK activation is involved in angiogenesis in several organs, *e.g.*, the placenta. Obesity before and/or during pregnancy (brown text and arrows) inhibit IGF-1 anti-inflammatory and mitochondrial protection activities, resulting in an increase in inflammation, ROS production and, hence, placental alterations. High-dense diets (red arrows) exacerbate GDM alterations, such as fetal overgrowth, macrosomia, fetal adiposity, inflammation, placental alterations and predisposition to disorders in adult’s life. ⊕ means enhancing protein activity.

Most IGF-1 actions are mediated through the union of this molecule to its putative receptor, IGF1R, an α_2_β_2_ heterotetrameric tyrosine kinase receptor (RTK), that activates the phosphatidylinositol-3-kinase (PI3K)/protein kinase B (AKT) (**Figure 1**) and mitogen-activated protein kinase (MAPK) signaling pathways, both related with cell survival, growth and proliferation (65). Due to its homology to proinsulin and IGF-2 structures (22), and the homology between receptors, IGF-1 can also bind to the insulin receptor (INSR) and IGF2R (a scavenger receptor homologous to manose-6-phosphate receptor), with lower affinity (65). Furthermore, IGF-1, IGF-2 and insulin can bind with lower affinity to hybrid receptors, conformed by one αβ-chain from INSR and another αβ-chain from IGF1R (65).

Both IGF-1 and IGF-2 are involved in cell survival and proliferation. Particularly, IGF-1 plays an essential role in modulating fetal growth due to its actions on mother and/or the placenta, *e.g.*, regulating nutrient supply and bioavailability (66). Moreover, IGF-1, *via* IGF1R and INSR downstream signaling pathways, participates in glucose transport to insulin sensitive tissues, such as skeletal muscle, adipose tissue and liver, decreasing glucose levels and improving insulin sensitivity, as IGF-1 levels does not oscillate over time as insulin does (67), thus reducing the hyperglycemic effect of GH (39). In normal pregnancies, placental hormones, *e.g.*, PL, progesterone, cortisol, GH and prolactin, can decrease the phosphorylation of insulin receptor substrate 1 (IRS-1), a key regulator of this signaling pathway, decreasing insulin sensitivity and β-cell function, leading to insulin resistance (68).

Clinical studies of GDM pregnancies have revealed an increase in maternal IGF-1 levels and a decrease in cord blood, and a positive correlation between insulin and IGF-1 fetal concentrations and birth weight of a newborn (69), suggesting the implication of this hormone in fetal intrauterine growth, that could lead to the development of macrosomia (70–72). Also, in GDM pregnancies, IGF-1 plays a crucial role in glucose homeostasis. Experimental and clinical studies have shown that placental insulin/IGF-1 pathway is promoted in GDM, as with energy-dense diets, increasing the activation of several downstream molecules, particularly mammalian target of rapamycin complex 1 (mTORC1), that augments nutrient transport across the placenta (73, 74) and regulates mitochondrial biogenesis and function (75, 76). In this way, mTORC1 activation could lead to fetal overgrowth, as it is positively correlated to birth weight (77–80). This mTORC1 activation could be a result of low circulating levels of adiponectin observed in the mother, a hormone that regulates glucose levels by inhibiting insulin/IGF-1 signaling pathway (76, 81) (**Figure 1**). In this sense, an inverse correlation between free IGF-1 and the risk of developing GDM has been found (13, 72).

IGF-1 activities must be rigorously controlled by its association with binding proteins (IGFBPs 1-6), found in several biological tissues and fluids, such as follicular liquid, amniotic liquid, vitreous humor, lymph, plasma, seminal fluid, cerebrospinal fluid and gastrointestinal secretions. However, the main source of IGFBPs is the liver (22). These binding proteins prolong the half-life in the circulation and modulate IGFs activities, due to their high affinity for both IGFs, rather than their own receptors (22). These binding proteins are capable of both inhibiting (*e.g.*, IGFBP-1) and enhancing (*e.g.*, IGFBP-3) IGFs biological activities, predominantly IGF-1. Recently, nine IGF-related binding proteins (IGFBP-rPs) have arose that can bind IGFs, but with lower affinity than IGFBPs (22).

IGFBP-3 is one of the principal proteins for IGF-1, as it regulates the bioavailability of this hormone. In the circulation, IGF-1 is found forming a ternary complex together with IGFBP-3 and ALS (acid-labile subunit) (22). It has been observed that increased concentrations of IGF-1 and IGF-1/IGFBP-3 molar ratio are related to an increased risk of GDM in early pregnancy (10-14 weeks of gestation) (82). Also, low cord serum levels of IGFBP-3 in GDM and obese women have been reported (83).

Another binding protein characterized by a high affinity for IGF-1, more than its own receptor, is IGFBP-1. This binding protein inhibits IGF-1´s biological action, thus reducing IGF-1 levels and preventing its downstream signaling pathway. IGFBP-1 is the principal binding protein in fetal circulation, whose production in the liver is inhibited by insulin (84) and food intake (85). In clinical studies, maternal obesity and GDM have been associated with an increase in IGF-1 levels, and low maternal and cord plasma levels of IGFBP-1, 3, 6 and IGFBP related protein 1 (IGFBPrP-1) (83, 86). These results hint that low serum and blood cord IGFBP-1 levels lead to an increase in IGF-1 bioavailability in GDM, probably due to the reduced phosphorylation of this binding protein observed in diabetes (84). This increase in IGF-1 accessibility produces the enlargement of the placenta and thus, an extra nutrient supply to the fetus, promoting fetal growth (84) and macrosomia, a characteristic detected in GDM pregnancies (70, 87). In this sense, IGFBP-1 levels are inversely correlated to fetal birthweight. Moreover, this decrease in IGBP-1 levels can be an outcome of increased fetal insulin secretion that inhibits the production of this binding protein (87).

Contrary to IGFBP-1, IGFBP-2, a binding protein that lacks postprandial fluctuation, has pleiotropic functions and is associated with glucose homeostasis (88). During early pregnancy (10-14 weeks of gestation), its levels are reduced and could function as an early marker of GDM risk (82). Clinical studies have shown decreased IGFBP-2 levels in both maternal and cord blood in GDM pregnancies (82, 87), leading to an augment of biologically active IGF-1 and IGF-2 that accelerates fetal growth.

Although most binging proteins have either inhibitory or enhancing functions, there are some that can have both roles. An example of this is IGFBP-4, the major substrate for the enzyme pregnancy-associated plasma protein-A (PAPP-A), a metalloproteinase that controls the bioavailability of IGFs. Particularly, PAPP-A modulates IGFs action through proteolysis of IGFBP-2, 4 and 5 (89), being expressed in several tissues. In the case of IGFBP-4, IGF-2 is a stronger facilitator of degradation then IGF-1 (90, 91), increasing in this way IGF-1 bioavailability and promoting cell growth (89). PAPP-A levels increase with the progress of gestation, as it is critical for trophoblast differentiation and invasion. Consequently, this enzyme has been employed as a diagnostic biomarker, especially during the first trimester, for several pregnancy disorders, such as Down syndrome (92). Experimental studies in macrophages and human preeclamptic placentas have shown that PAPP-A overactivation activates PI3K/AKT signaling pathway, producing an inflammatory response (93–96), that could lead to endothelial dysfunction, a common feature of both preeclampsia and GDM (**Figure 1**).

IGFBPs not only serve to control the bioavailability and activities of IGFs. IGFBP-1 and 2 have IGF-independent effects, as they can bind to α_5_β_1_-integrin receptors and activate the PI3K/AKT signaling pathway, triggering several molecular targets, *e.g.*, focal adhesion kinase (FAK), involved in glucose uptake and insulin sensitivity (67, 97–99). This molecular cascade activated *via* FAK, as shown in cell cultures of extravillous trophoblasts (100), it is also involved in focal adhesions and cell motility *via* both PI3K/AKT and MAPK signaling pathways (67, 101, 102), embroiled in developing an adequate placentation. Another ligand that bind to integrins is the soluble form of the vascular endothelial growth factor receptor 1 (sVEGFR-1 or fms-like tyrosine kinase receptor-1, sFlt-1), a decoy receptor for vascular endothelial growth factor A (VEGF-A). It decreases angiogenesis at the embryogenesis stage *via* vascular endothelial growth factor receptor 2 (VEGFR-2) signaling (103–105). sVEGFR-1 is a key regulator of the formation of new blood vessels during embryogenesis. Mutant mice for this receptor die at this stage due to an abnormal growth and dysfunction of blood vasculature (106). Also, the overexpression of sVEGFR-1 in human placentas alters angiogenesis and results in endothelial dysfunction (37, 107–109), due to the impairment of signal transduction through VEGF-A (110–113). This adverse circumstance, could result in hypervascularization of the placenta, that lead to numerous physiological alterations in this organ, such as villous immaturity, villous fibrinoid necrosis and chorangiosis, as it is observed in GDM pregnancies (114). This reveals the increasing oxygen demand of the fetus, due to the insulin-stimulated enhanced fetal aerobic metabolism (115, 116) (**Figure 1**). Moreover, studies in human placentas showed decreased VEGFR-1 (mRNA and protein) and VEGFR-2 levels (mRNA) (115, 117), while reports in human umbilical vein endothelial cells (HUVECs) disclosed that GDM enhanced cell migration (115), suggesting that GDM promotes an angiogenic state that could affect the pathophysiological function of the placenta.

## Conclusion

IGF-1 bioavailability is one of the main discordant factors for the development of GDM during pregnancy, this is where IGFBPs, especially IGFBP-1 and IGFBP-2, play a significant role. Obesity, both before and/or during pregnancy, a condition related to consumption of energy-dense diets, can alter IGF-1 secretion and actions, leading to GDM. Obesity also decreases the levels of these IGFBPs, thus increasing the bioavailability of IGF-1, promoting an increase of nutrient availability to the fetus, which can lead to overgrowth and other metabolic complications, characteristics of GDM. Similarly, there are other molecules capable of exacerbating the adverse effects of GDM, such as sVEGFR-1, which activates FAK, a protein also indirectly involved in the IGF-1 signaling pathway, β-cell exhaustion and placental hypervascularization. Therefore, knowledge of the molecular targets of IGF-1 and their interaction with other molecules involved in several important cellular processes during pregnancy, *e.g.*, placental angiogenesis, are a good starting point to develop new therapeutic targets. This could lead to a better quality of life in patients and, in this case, newborns, reversing or even preventing the development of metabolic diseases in adulthood, which would have serious consequences for their health.

## Author Contributions

All authors participated directly in the manuscript. IM-E: investigation, writing-review and digital art; FC-T: conceptualization, investigation and writing-review. All authors have read and agreed to the published version of the manuscript.

## Conflict of Interest

The authors declare that the research was conducted in the absence of any commercial or financial relationships that could be construed as a potential conflict of interest.

## Publisher’s Note

All claims expressed in this article are solely those of the authors and do not necessarily represent those of their affiliated organizations, or those of the publisher, the editors and the reviewers. Any product that may be evaluated in this article, or claim that may be made by its manufacturer, is not guaranteed or endorsed by the publisher.

## Abbreviations

AKT, protein kinase B; ALS, acid-labile subunit; FAK, focal adhesion kinase; FGR, fetal growth restriction; GDM, gestational diabetes mellitus; GH, growth hormone; GHRH, growth hormone-releasing hormone; HUVECs, human umbilical vein endothelial cells; IGFs, insulin-like growth factors; IGF-1, insulin-like growth factor 1; IGF1R, IGF-1 receptor; IGF-2, insulin-like growth factor 2; IGF2R, IGF-2 receptor; IGFBPs, IGF binding proteins; IGFBP-rPs, IGFBP-related protein 1; INSR, insulin receptor; IRS-1, insulin receptor substrate 1; HOMA-IR, Homeostatic Model Assessment for Insulin Resistance; MAPK, mitogen-activated protein kinase; mTORC1, mammalian target of rapamycin complex 1; NO, nitric oxide; PAPP-A, enzyme pregnancy-associated plasma protein-A; PI3K, phosphatidylinositol-3-kinase; PL, placental lactogen; RTK, tyrosine kinase receptor; sFlt-1, fms-like tyrosine kinase receptor-1; T2DM, type 2 diabetes mellitus; VEGF, vascular endothelial growth factor; VEGF-A, vascular endothelial growth factor A; sVEGFR-1, soluble form of the vascular endothelial growth factor receptor 1; VEGFR-2, vascular endothelial growth factor receptor 2; WHO, World Health Organization.
